# Bacterial and parasite co-infection in Mexican golden trout (*Oncorhynchus chrysogaster*) by *Aeromonas bestiarum*, *Aeromonas sobria*, *Plesiomonas shigelloides* and *Ichthyobodo necator*

**DOI:** 10.1186/s12917-022-03208-5

**Published:** 2022-04-12

**Authors:** María Anel Fuentes-Valencia, José Luis Osornio-Esquivel, Carlos Antonio Martínez Palacios, José Luis Contreras-Ávila, Erik Barriga-Tovar, Genoveva Ingle-de la Mora, Andrés Arellano-Torres, Víctor Manuel Baizabal-Aguirre, Alejandro Bravo-Patiño, Marcos Cajero-Juárez, Juan José Valdez Alarcón

**Affiliations:** 1 Centro Multidisciplinario de Estudios en Biotecnología, Facultad de Medicina Veterinaria y Zootecnia, Universidad Michoacana de San Nicolás de Hidalgo, Morelia, Mexico; 2 Instituto de Investigaciones Agropecuarias y Forestales, Universidad Michoacana de San Nicolás de Hidalgo, Morelia, Mexico; 3 Comité Estatal de Sanidad e Inocuidad Acuícola de Michoacán A.C. (CESAMICH), Morelia, Mexico; 4 Dirección General Adjunta de Investigación en Acuacultura, Instituto Nacional de Pesca y Acuacultura, Pátzcuaro, Mexico; 5 Centro Regional de Investigación Acuícola y Pesquera en Pátzcuaro, Instituto Nacional de Pesca y Acuacultura, Pátzcuaro, Mexico

**Keywords:** Salmonids, *Oncorhynchus chrysogaster*, Bacterial coinfection, Antimicrobial resistance

## Abstract

**Background:**

Bacterial infections are responsible of high economic losses in aquaculture. Mexican golden trout (*Oncorhynchus chrysogaster*) is a threatened native trout species that has been introduced in aquaculture both for species conservation and breeding for production and for which no studies of bacterial infections have been reported.

**Case presentation:**

Fish from juvenile stages of Mexican golden trout showed an infectious outbreak in a farm in co-culture with rainbow trout (*Oncorhynchus mykiss*), showing external puntiform red lesions around the mouth and caudal pedunculus resembling furuncles by *Aeromonas* spp. and causing an accumulated mortality of 91%. Isolation and molecular identification of bacteria from lesions and internal organs showed the presence of *Aeromonas bestiarum*, *Aeromonas sobria*, *Plesiomonas shigelloides* and *Ichthyobodo necator* isolated from a single individual. All bacterial isolates were resistant to amoxicillin-clavulanic acid and cefazoline. *P. shigelloides* was resistant to third generation *β*-lactamics.

**Conclusions:**

This is the first report of coinfection by *Aeromonas bestiarum*, *Aeromonas sobria*, *Plesiomonas shigelloides* and *Ichthyobodo necator* in an individual of Mexican golden trout in co-culture with rainbow trout. Resistance to *β*-lactams suggests the acquisition of genetic determinants from water contamination by human- or livestock-associated activities.

## Highlights

1. This is the first report of a coinfection by *Aeromonas bestiarum*, *Aeromonas sobria*, *Plesiomonas shigelloides* and the ectoparasite *Ichthyobodo necator* in a Mexican golden trout (*Oncorynchus chrysogaster*), a threatened native species.
2. The antibiotic resistance profiles suggest the influence of the water source contaminated by human activities.

## Background

Mexico has a high diversity of endemic trout species which are considered the most southerly salmonids compared to the natural distribution of other salmonids [1–3]. Mexican golden trout (*Oncorhynchus chrysogaster*) is a native species living at heights greater than 1900 m over the sea level, in the basins of the Sinaloa, Culiacán and El Fuerte rivers in the Sierra Madre Occidental in México. Mexican golden trout is the most important source of food protein for surrounding human populations [1, 4]. Mexican golden trout is considered either a threatened or a near threatened native species by International Union for the Conservation of Nature and Natural Resources (IUCN) [4] or national NOM-059-SEMARNAT-2010 [5] regulatory organisms, respectively. The threatened condition is due to deforestation, habitat degradation, climate change and overexploitation [2]. Another threat for Mexican golden trout is the current hybridization with an exotic salmonid, the rainbow trout (*Oncorynchus mykiss*) which is considered one of the more harmful to native fish exotic species [4, 6].

The genetic background of native trout provides this species with a unique adaptation to environment that are not favourable to other salmonids, so commercial breeding programs have been proposed [1, 2] to avoid loss of genetic background for the native species [7, 8]. Important threats for aquaculture are also illnesses; accounting for 50% of the decrease in production, being those caused by bacteria the most significant [9, 10].

*Aeromonas* spp. are important pathogens for aquaculture [11]. They are Gram-negative, oxidase positive bacilli [12]. All species but *A. media* and *A*. *salmonicida* are motil due to the presence of a polar flagellum and all are ubiquitous of brackish water and freshwaters [13]. *A. salmonicida, A. hydrophila*, *A. caviae*, *A. veronii*biovar *sobria,A. veronii* biovar *veronii*, *A. dhakensis,A. encheleia, A. allosaccharophila,A. schubertii*, *A. bestiarum*, *A. sobria*, *A. piscicola* and *A. jandaei*have been reported as pathogens in fish culture [14–16]. Particularly important are *A. hydrophila*, *A. caviae* and *A. veronii* which cause sepsis and ulcerative syndrome with a high economic impact on production [17]. *A. salmonicida* has been recognized as the causal agent of the so-called “Ulcer disease” or “Red-Sore disease” [18] which may reach mortalities to about 90% in fish farms [19]. To date, there are no reports of *Aeromonas* spp.-related disease in Mexican golden trout.

*Plesiomonas shigelloides* is also a Gram-negative, oxidase positive, motile Enterobacteriaceae [20]. Along with *Aeromonas* spp. and *Fusobacterium mortiferum*, they are common pathogens in the gastrointestinal tract of freshwater fish from orders Perciformes (*Micropterus salmoides*, *Lepomi macrochirus*), Siluriformes (*Hypostomus auroguttatus, Ictalurus punctatus, Pimeolodus maculatus*), Salmoniformes (*O. mykiss*) and Characiformes (*Prochilodus argenteus*) among others [21–23]. In rainbow trout (*Oncorhynchus mykiss*) *P. shigelloides* infections has been associated with thin, weak, 1-2 year old fish showing yellowish exudate from anus, petechiae and ascites in internal organs and 40% mortality [24]. In grass carp (*Ctenopharyngodon idellus*) *P. shigelloides* causes muscular erosion [25] while in silver carp (*Hypophthalmichthys molitrix*) 60% mortality showing exophthalmia and diffuse haemorrhagic spots [26]. In ornamental cichlids *P. shigelloides* may cause up to 100% mortality [27].

Infestations by parasites from the genus *Ichthyobodo*, have been reported in salmonids from the genus *Oncorhynchus* [9, 28–33]. In particular, *Ichthyobodo necator* can cause mortalities of up to 40% [28, 32]. Fish showing Ichthyobodosis usually show a grayish layer over the skin, loss of epidermis and small ulcers, which have been related to secondary infections [34]. The goal of the present study was to identify bacteria associated with an apparent outbreak of Red-Sore disease in a farm cultivating both Mexican golden trout and rainbow trout.

## Case presentation

### Case history

**Fig. 1:**
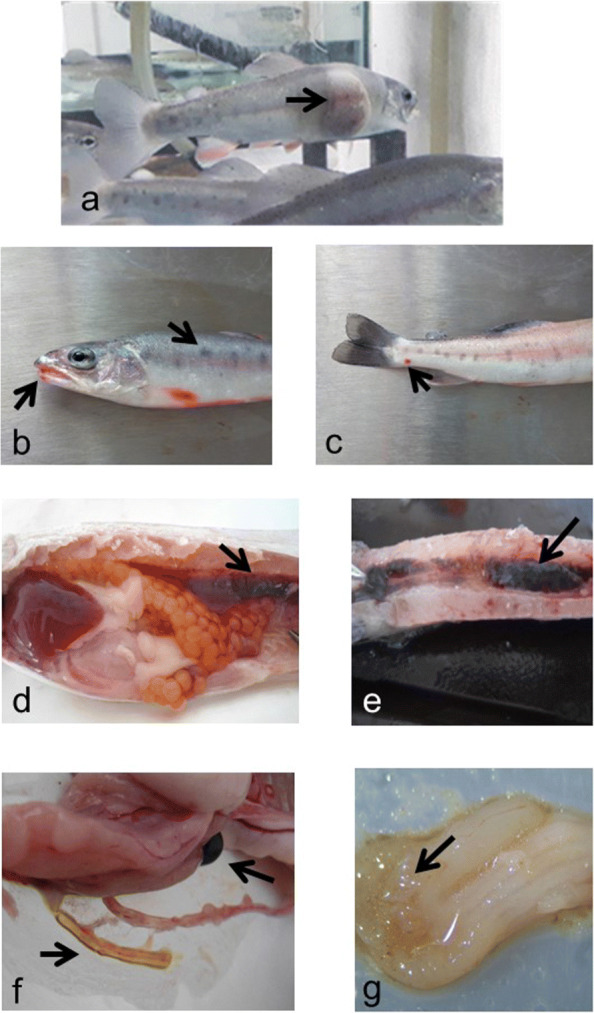
Lesions in Mexican golden trout. External lesions: **a** Ulcer and grayish layer over the skin; **b** Mouth lesion and scale loss; **c** Skin ulcer in peduncle. Internal lesions. **d** Kidney inflammation with abnormal firmness; **e** necrotic kidney; **f** gelatinous yellow faeces and spleen presenting black spots; **g** stomach with low food content Arrows point to the lesions

In a rainbow trout (*Oncorhynchus mykiss*) production farm in the municipality of Pucuato, in the State of Michoacán de Ocampo, México, located at 19^∘^39’N, 100^∘^45’O and 2,511 m.a.s.l., Mexican golden trout (*Oncorhynchus chrysogaster*) is also cultivated as an experimental approach for domestication and farming. Rainbow trout is cultivated in concrete tanks fed with water from the “El Retranque” dam in Pucuato. This facility also contains an experimental area with PVC gutters and glass aquariums. The water source for this area is from a spring shared with the nearest human settlement in the town of Pucuato. In September, 2019, the experimental Mexican golden trout showed an outbreak of an infectious disease with a duration of 35 days that caused 91% of accumulated mortality, with 12 of 131 individuals surviving. On the 33rd day, 6 specimens were collected with mean lengths and weighs of 11.33 cm and 53.9 g, respectively. The specimens were transported to the laboratory alive for clinical descriptions of pathological signs and microbiological analysis. Specimens showed normal swimming with preferent location at the borders of the container. Red and inflamed lesions were observed in the abdomen and mouth, fins were haemorrhagic and gills were inflamed with petechiae (Fig. 1). An ectoparasite, *Ichthyobodo necator*, was identified from fresh samples of skin and gills. Stomachs showed low content of chyme. The bowel showed either soft brown (4 individuals) or gelatinous yellow (2 individuals) faeces. Gallbladders were yellow. Spleens with black spots, abnormal firmness and presence of fat. Kidneys showed mild inflammation, abnormal firmness and in one individual it was necrotic. For microbiological analysis, samples from skin, inflamed fins, gelatinous faeces, kidney, spleen, brain, heart and gallbladder were collected with a cotton swab [35] and plated in rich (Soybean Trypticase, Brain-Heart Infusion) and selective (McConkey, *Salmonella-Shigella*, Cefsulodin-irgasan-novobiocin – CIN -, Cetrimide, Glutamate-starch-phenol red – GSP - and Sabourad) solid culture media. Isolates were obtained from rich media and GSP agar which is a selective medium for *Aeromonas* spp. Since signs were similar to Red Sore Disease, we proceed to analyse only GSP agar isolates for the search of *Aeromonas* spp.

At the 3^*rd*^,5^*th*^ and 7^*th*^ day of the beginning of the outbreak, the volume of water in the tanks was reduced to 50% and 25 ml/l of 10% of commercial aldehyde product (Paraguard, Seachem) were added. On day 8 and 10, 25mg/l of kanamycin sulfate (Kanaplex, Seachem) was applied. From days 12^*th*^ to 16^*th*^, 20 mg/l of a mixture of Sulfamethoxypyridacin (125 mg), Trimethoprim (25 mg) and tylosine (30 mg) per g of product (Koryn Triple, Tornel). On days 17 to 19, Kanaplex was applied again but mixed with 10 g/l NaCl. From days 21 to 28, oxytetracycline (20 mg/kg of fish weight) was applied until mortality stopped. Florfenicol (Florfen 10, Preveson) 10 mg/kg of live weight was then incorporated in the food for 6 additional days, until fish do not take the food anymore.

**Fig. 2:**
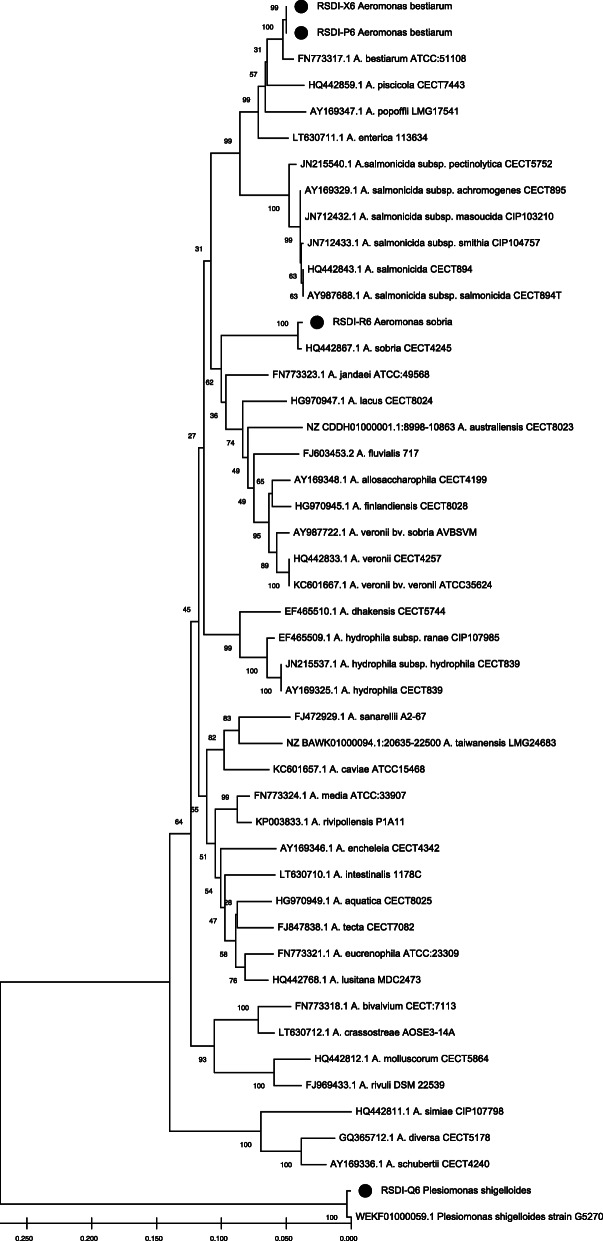
Similarity tree of bacterial isolates. Black dots represent the position in the tree of the isolates reported in this work. Numbers in each node are the confidence values. Distance bar is the number of nucleotide substitutions per site

Bacterial isolation was performed as described in Whitman et al. (2004). Liver, spleen and kidney from five individuals with Red Sore disease signs were aseptically collected and samples were inoculated in GSP culture medium which is selective for the genus *Aeromonas*. Yellow colonies formed by Gram-negative oxidase positive bacilli were further analysed for motility and glucose fermentation. Isolates that fit to *Aeromonas* spp. phenotype were stored in Brain-Heart infusion (BHI) added with 15% glycerol at -75^∘^C for further characterization. Molecular identification of presumptive *Aeromonas* spp. isolates was performed by sequencing an 820 bp fragment from *rpoD* gene [36]. Sequences were obtained from Macrogen (Korea) and assembled with Unipro UGENE v39.0 sequence assembly software v5.15 [37]. Sequences were registered under GenBank numbers MZ668583 (RSDI-X6), MZ668586 (RSDI-P6), MZ668585 (RSDI-R6) and MZ668584 (RSDI-Q6). Similarity search was performed with Basic Local Alignment Research Tool (BLAST) at the National Center of Biotechnology Information (NCBI, USA). Four bacterial isolates were obtained. Two of them correspond to *Aeromonas bestiarum* isolated from oral lesion (RSDI-X6) and spleen (RSDI-P6) with 99.27% and 99.05% of sequence identity for each sequence. One isolate (RSDI-R6) sequence was 98.61% identical to *Aeromonas sobria* isolated from kidney, and the last one (RSDI-Q6) showed also 99.29% identity with a *P. shigelloides* sequence and was isolated from spleen. Isolates RSDI-P6, RSDI-R6 and RSDI-Q6 were recovered from the same individual. To assess evolutionary relatedness of the bacterial species, a similarity tree (Fig. 2) was constructed using MEGA (Molecular Evolutionary Genetics Analysis) version X software [38]. Evolutionary relatedness was inferred using the Neighbour joining method [39]. Confidence values were estimated with a bootstrap test with 1000 replicates [40] and evolutionary distances with the Kimura-2 parameter method [41]. Figure 2 shows strong evolutionary relatedness of our *A.bestiarum* (RSDI-X6, RSDI-P6, 100% of confidence) and *A. sobria*(RSDI-R6, 100% of confidence) isolates with reference sequences. Isolate RSDI-Q6 identified as *P. shigelloides*, also associated with a reference sequence with 100% of confidence.

Antimicrobial sensitivity tests were performed according to the Clinical and Laboratory Standards Institute (CLSI, USA) manuals M45 and M100 for disk diffusion tests [42, 43]. Isolates were tested against 21 antibiotics at their specified concentration (Table 1). Briefly, 24 h cultures at 28^∘^C were adjusted to an OD_610*nm*_ of 0.5-0.7 and plated with a cotton swab in Muller-Hinton agar over which sensidiscs (Oxoid) were placed. After 24h, the inhibition zone was measured with a Vernier calibrator. The three *Aeromonas* spp. isolates were resistant or intermediate resistant to Amoxicillin-clavulanic acid, ampicillin-sulbactam, cefazolin and cefoxitin. RSDI-X6 and RSDI-P6 *A. bestiarum* isolates were resistant to piperacillin-tazobactam while *A. sobria* (RSDI-R6) showed intermediate susceptibility. The three *Aeromonas* spp. Isolates were resistant to carbapenems (ertapenem, imipenem, meropenem). *Aeromonas* spp. Isolates were sensitive to cefepime, cefotaxime, ceftazidime, ceftriaxone, cefuroxime sodium, aztreonam, tetracycline, ciprofloxacin, levofloxacin, trimethoprim-sulfamethoxazole and chloramphenicol.

**Table 1:** Antibiotic sensitivity test results

Antimicrobial agent	Concentration (*μ*g)	Isolates			
		RSDI-X6 *Aeromonas bestiarum*	RSDI-R6 *Aeromonas sobria*	RSDI-P6 *Aeromonas bestiarum*	RSDI-Q6 *Plesiomonas shigelloides*
		Interpretative critera of sensitivity			
Penicillins in combination with other *β*-lactamics / *β*-lactamase inhibitors					
Amoxicillin- clavulanic acid	AMC 20/10	R	R	R	I
Ampicillin – sulbactam	SAM 10/10	R	R	R	S
Piperacillin – tazobactam	TZP 100/10	R	I	R	S
Cephalosporins					
Cefazolin	KZ 30	R	R	R	R
Cefepime	FEP 30	S	S	S	S
Cefotaxime	CTX 30	S	S	S	R
Cefoxitin	FOX 30	R	R	R	S
Ceftazidime	CAZ 30	S	S	S	I
Ceftriaxone	CRO 30	S	S	S	S
Cefuroxime sodium	CXM 30	S	S	S	S
Carbapenems					
Ertapanem	ETP 10	R	R	R	S
Imipenem	IPM 10	R	R	R	I
Meropenem	MEM 10	R	R	R	R
Monobactams					
Aztreonam	AZT30	S	S	S	S
Aminoglycosides					
Amikacin	AK 30	S	S	I	R
Gentamicin	CN 10	S	I	I	R
Tetracyclines					
Tetracycline	TE 30	S	S	S	S
Quinolones					
Ciprofloxacin	CIP 5	S	S	S	I
Levofloxacin	LEV 5	S	S	S	S
Folate pathway inhibitors					
Trimethoprim/sulfamethoxazole	SXT 1.25/23.75	S	S	S	S
Phenicols					
Chloramphenicol	C 30	S	S	S	S

*P. shigelloides* (RSDI-Q6) isolate showed resistance to cefazolin, cefotaxime, meropenem, amikacin, gentamicin, intermediate resistance to amoxicillin-clavulanic acid, ceftazidime and meropenem, and was sensitive to the rest of the antibiotics.

For the identification of ectoparasites, wet mount examination of gill clips and skin scrapes taken from several sites on the fish were examined by light microscopy. *I. necator*was identified in skins and gills samples. Due to the high mortality rate and the late moment in which samples were collected for analysis, it was not possible to analyse healthy fish from this outbreak for the search of ectoparasites. However, in august 2018, in sick rainbow trouts from the same farm, *A. hydrophila*, *A. sobria*, or *A. allosaccharophyla* were isolated from internal organs, but no ectoparasites were observed. In December 2018, neither of the above-mentioned bacteria nor ectoparasites, were found in healthy fish from the same farm.

## Discussion and conclusions

Although Mexican golden trout is an endangered species ongoing transition to commercial exploitation, no research is available on its bacterial pathogens. This is the first report of coinfection of Mexican golden trout with *A*. *bestiarum*, *A*. *sobria*, *P. shigelloides* and the ectoparasite *Ichthyobodo necator*.

*I. necator* is a common parasite of the genus *Oncorhynchus*, infesting species such as *O. mykiss*, *O. tshawytscha*, *O. masou*, *O. gorbuscha*, *O. keta*y *O. nerka* [9, 28–33]. In this report, *A. bestiarum* RSDI-X6 was isolated from an external lesion. Epithelial destruction by ectoparasites causes an imbalance in osmoregulation followed by hyperplasia and lamellar fusion of the gills followed by respiratory malfunction [28, 44]. Synergistic effects of ectoparasites and bacteria co-infecting salmonids, have been described [45–47], but has also been suggested that ectoparasites may act as vectors of bacteria in fish infections [48–50]. Further studies are necessary to demonstrate that *I. necator* has a role as a vector of bacterial infections in Mexican golden trout.

*Aeromonas* spp. are well known to cause high mortality in salmonids [18, 51].In the aquatic environment, fish are exposed to a great variety of pathogens [52] with co-infections as a factor increasing susceptibility [53]. Co-infections can be a challenge for diagnosis and treatment [52]. To the best of our knowledge, there is only a report on bacterial co-infections for the genus *Oncorhynchus*. In a study of pathogen prevalence in Chinook salmon (*O. tshawytscha*) several bacterial pathogens were identified, but a significant association was only observed for *Renibacterium salmoninarum* and aeromonads; *R. salmoninarum* and *A. salmonicida* were the most abundant pathogens with prevalence values about 25% and 15%, respectively [54]. Bacterial co-infections are common in other fish species in aquaculture and may alter prevalence, severity and impact on fish disease or success of vaccination strategies due to the synergistic effect of the pathogens [53]. In Nile tilapia (*Oreochromis niloticus*), co-infections have been described with *Streptococcus agalactiae, Streptococcus iniae, Francisella noatunensis*subsp. *orientalis, A. hydrophila, P. shigelloides*and *Edwardsiella tarda* [55].

*A. bestiarum, A. sobria*and *P. shigelloides* identified in Mexican golden trout in this work, are common inhabitants of aquatic ecosystems. *Aeromonas spp.* and *P. shigelloides*are also considered as emergent pathogens of intestinal and extraintestinal diseases [56–58]. *Aeromonas* spp. and *P. shigelloides* were previously isolated from fish, amphibians, molluscs, crustaceans, reptiles and mammals [12, 59–61]. *Aeromonas* spp. have been proposed as indicators of the presence and development of microbial resistance to antibiotics both in fish farms and natural environment, and as a potential source of transmission of resistance determinants to human pathogens, as they are zoonotic [58, 62]. *Aeromonas* spp. are also commonly found in vegetables, foods of animal origin, faeces of animal and human origin and contaminated water as important sources of transmission to humans [63]. In intestinal outbreaks, frozen foods or insufficiently cooked meals have also been associated with *Aeromonas* spp. transmission [64]. In some foods, *Aeromonas* spp. cell densities may reach up to 10^5^ bacteria ·g^−1^ or ml^−1^ [57]. Due to the presence of antimicrobial resistance, virulence and biofilm producing gene markers in isolates from aquaculture and abattoir environments, *Aeromonas* spp. are becoming good indicators of water and food quality [65, 66].

*P. shigelloides*, previously classified as *Aeromonas shigelloides*, share several features with *Aeromonas* spp. It is also commonly found in foods of animal and plant origin [57]. *P. shigelloides* has high clinical relevance because it has been ranked as the third and fourth causes of gastroenteritis in Nigeria and China, respectively [67, 68], being water its most common source of transmission [64]. *P. shigelloides* may grow easily in a great variety of foods [57]. Ingestion of seafoods as oysters and uncooked fish meals have been associated with outbreaks of *P. shigelloides* infections [64]. So, since *Aeromonas* spp. and *P. shigelloides* are both considered as good indicators of food contamination and as emergent pathogens in aquaculture, the finding of these two pathogens in co-infection in an outbreak in Mexican golden trout suggest external contamination of water sources.

In this report three of the *Aeromonas* spp. isolates from Mexican golden trout were resistant to *β*-lactamic antibiotics, particularly to first and second generation cephalosporins (cefazolin and cefoxitin) and *β*-lactamase inhibitors (clavulanic acid and sulbactam). Amoxicillin and ampicillin resistance were also reported for rainbow trout (*O. mykiss*), tilapia (*O. mossambicus*) and Koi carp (*Cyprinus carpio*) *Aeromonas* spp. isolates in South Africa [69]. Resistance to *β*-lactams have been increasing in *Aeromonas* spp. from clinical and environmental origin [70, 71]. In México, *Aeromonas* spp. isolates from rainbow trout contained extended spectrum *β*-lactamases (ESBL) encoding genes (*bla*_*SHV*_ y *bla*_*CphA*/*IMIS*_) [72]. *Aeromonas* spp. isolates from this report were susceptible to third generation cephalosporins and monobactam, suggesting the absence of ESBL gene determinants, as has been reported for trout, tilapia and Koi carp isolates [69]. Susceptibility to monobactams, tetracyclines, quinolones and phenicols in our *Aeromonas* spp. isolates are in accordance with a previous study [12].

Our *Plesiomonas shigelloides*isolate showed resistance and intermediate resistance to cefotaxime, ceftazidime respectively, third generation cephalosporins for which ESBL bacteria are resistant. ESBLs are common in *Escherichia coli*and other Enterobacteriaceae [73], which were found in isolates from intestinal samples in fish from India and Switzerland [74, 75]. Since third generation cephalosporins are not commonly recommended for treatment of bacterial infections in aquaculture, it is possible that ESBL genetic determinants may be mobilizing from contaminating enterobacteria from the environment that are horizontally transferring their genes to *P. shigelloides*. High prevalence of cephalosporin resistant *P. shigelloides* suggest also high level of contamination of water [76], although isolates from other sources (humans, dogs, aquarium) were susceptible to cephalosporins [77]. *P. shigelloides* isolate RSDI-Q6 also showed intermediate resistance to *β*-lactams (amoxicillin-clavulanic acid) and a first-generation cephalosporin (cefazolin). Resistance to a wide variety of *β*-lactams in *P. shigelloides*isolates is common [78]. This result is in contrast to that reported for *P. shigelloides* isolates from tilapia which were susceptible to amoxicillin-clavulanic acid, ceftazidime and gentamicin [79]. These same authors also reported resistance to tetracycline and chloramphenicol, for which our isolate was susceptible. *Aeromonas* spp. and *P. shigelloides* isolates in this work were either resistant or intermediate-resistant to amoxicillin-clavulanic acid and cefazolin, a *β*-lactam/ *β*-lactamase inhibitor, a first-generation cephalosporin and carbapenems. Neither of these antibiotics are approved by the Food and Drug Administration from USA which are also applied in Mexican regulations, so genetic resistance may be acquired from other bacterial species from animal or human origin contaminating the water. Although it has been suggested that there may be some intrinsic resistance to these antibiotics for these species [79]; evidence is also reported that describe sensitive and resistant isolates both from clinical and environmental origin [80] for *Aeromonas* spp. It is suggested that variation in antimicrobial susceptibility may be due to different genetic backgrounds in the environment and the selective pressures by antibiotics contaminating the water sources [70], reinforcing the hypothesis that antibiotic resistance determinants in bacteria from aquatic environments may be a consequence of anthropogenic contamination [62]. The presence of *Aeromonas* spp. showing antibiotic resistance may be used as indicators of contamination in vulnerable aquatic environments [81]. According to this, presence of integrons and other mobile genetic elements are frequently found that encode antibiotic resistance determinants in *Aeromonas* spp. [82]. Antibiotics are commonly used in anthropogenic activities related to aquaculture, animal production and even in treatment of companion animals, so its presence is frequent in the environment and they become a risk for public health [83, 84]. Resistance to antibiotics not commonly used in aquaculture is also frequent in other fish pathogenic bacteria [85]. Antimicrobial resistance is considered an important health risk in aquaculture [86], particularly for Mexican golden trout and their cross breeds due to their threatened condition. This particular threat for Mexican golden trout is added to others as geographic isolation, habitat transformation, chemical contamination from pesticides and industrial and mining activities in the surrounding environment [87, 88].

This report describes the co-infection of *Aeromonas* spp. and *P. shigelloides* in a Mexican golden trout operation and outlines their antibiotic resistance to *β*-lactams and third generation cephalosporines which suggests an infection from contaminated waters. Due to the emerging importance of these bacterial species as environmental quality markers and as emergent zoonotic pathogens, it is important to make a continuous surveillance of these pathogens in aquaculture. To complement surveillance, it is necessary to perform studies on the presence of virulence factors, haemagglutination patterns, infectivity in cellular models, and biofilm forming abilities, among others, to better understand the pathogenic potential of each isolate. Studies with multilocus sequence typing (MLST) [89] and macro-restriction analysis in pulse-field gel electrophoresis (PFGE) [90] will also help to understand Aeromonads pathogen diversity and the possible relation of particular clones as human or fish pathogens or as environmental strains [91]. Comparative genome analysis of our *Aeromonas* spp. and *P. shigelloides* isolates against others from different sources will also allow understanding differences in pathogenic potential and host specificity and how are they contributing to the co-infection process.

## Data Availability

All data related to this study are included in this manuscript. Sequences obtained in the present study are available in the GenBank repository (https://www.ncbi.nlm.nih.gov/nuccore) under accession numbers: MZ668583 (RSDI-X6), MZ668586 (RSDI-P6), MZ668585 (RSDI-R6) and MZ668584 (RSDI-Q6).

## Abbreviations

GSP: Glutamate Starch Phenol red
BHI: Brain-heart infusion
BLAST: Basic Local Alignment Search Tool
NCBI: National Center for Biotechnology Information
CLSI: Clinical and Laboratory Standards Institute
FDA: Food and Drug Administration
IUCN: International Union for Conservation of Nature and Natural Resources
ESBL: Extended-spectrum *β*-lactamases
